# Efficient Inhibition of *Streptococcus agalactiae* by AIEgen-Based Fluorescent Nanomaterials

**DOI:** 10.3389/fchem.2021.715565

**Published:** 2021-07-20

**Authors:** Mengmeng Yi, He Wang, Miao Wang, Jianmeng Cao, Fengying Gao, Xiaoli Ke, Zhigang Liu, Ying Liu, Maixin Lu

**Affiliations:** ^1^ Key Laboratory of Tropical and Subtropical Fishery Resource Application and Cultivation, Ministry of Agriculture, Guangdong Provincial Key Laboratory of Aquatic Animal Immune Technology, Pearl River Fisheries Research Institute, Chinese Academy of Fishery Science, Guangzhou, China; ^2^ Key Laboratory of Environment Controlled Aquaculture, Ministry of Education, Dalian Ocean University, Dalian, China

**Keywords:** aggregation induced emission, TBP-1, *Streptococcus agalactiae*, antimicrobial activity, ROS

## Abstract

*Streptococcus agalactiae*, referred to as group B streptococcus (GBS), is a prominent co-pathogenic bacterium causing the onset and death of human, animal, and aquatic products. Although antibiotics are efficient against GBS, antibiotic resistance through antibiotic overuse is an equally serious problem. Therefore, the treatment of GBS infection appears strongly dependent on nonantibiotic therapy, such as photodynamic therapy. Different from other photosensitizers (PSs), luminogens with aggregation-induced emission (AIEgen) can efficiently generate fluorescence and reactive oxygen species (ROS). Herein, TBP-1, an efficient AIE PSs, is chosen to resist GBS, and its antibacterial activity and the killing mechanism toward GBS are investigated. The ROS generation performance and the images of GBS treated with TBP-1 in the dark or under white light irradiation were investigated. TBP-1 with its high ROS generation ability can efficiently kill GBS and serve as a novel treatment strategy against GBS infection.

## Introduction

*Streptococcus agalactiae*, referred to as group B streptococcus (GBS), is a prominent co-pathogenic bacterium causing the onset and death of human, animal, and aquatic products. GBS is pervasive in nature and can colonize in the digestive and urogenital systems of the organism. GBS causes severe infectious diseases in humans with pathetic immunity, such as immunocompromised patients, aged, neonates, and pregnant women (including bacteremia/sepsis, infectious endocarditis, septic arthritis, and meningitis) (Hall et al., 2017; Takahashi et al., 2010; Takahashi et al., 2011). GBS can also be isolated from dairy cows, in which it causes clinical mastitis, resulting in a significant economic loss (IngridHolmøy et al., 2019; Sørensen et al., 2019). In addition, GBS is a prominent and ubiquitous pathogen in aquaculture, causing very high morbidity (generally 20–30%) and mortality (over 95% of diseased fish), especially in the tilapia aquaculture industry, around the world annually (Su et al., 2017). Although GBS is sensitive to antibiotics, such as penicillin and cephalosporin (Moroi et al., 2019; Seki et al., 2015), antibiotic resistance through antibiotic overuse is an equally serious problem (Alejandro et al., 2021). Therefore, GBS has been recognized as one of the most significant public health threats at present (Caruso et al., 2021). According to the Centers for Disease Control and Prevention (CDC), in the United States, the annual mortality is about 1.15% of those infected with antibiotic-resistant bacteria; this fully certifies the gravity of the situation (Thakur et al., 2021).

Therefore, the treatment of this pathogenic bacterial infection appears to be strongly dependent on nonantibiotic therapy, such as photodynamic therapy (Zhou et al., 2018). However, inefficient ROS induced by conventional photosensitizers seriously restricts their extensive applications in therapy of pathogenic infection due to aggregation (Hu et al., 2018). Aggregation-induced emission (AIEgen) luminogen is a promising approach for therapy as well as diagnosis since both fluorescence and ROS generation performance of AIEgen are enhanced with aggregation (Chen et al., 2018). Previous research has certified that different AIEgen families are effective for antibacterial activity (Zhao et al., 2015; Shi et al., 2019; Shi et al., 2020a); however, inhibiting GBS by AIEgen underlying mechanisms have not been investigated yet.

In this study, TBP-1 was chosen to explore its ability against GBS since TBP with both visible spectrum absorption and fluorescence emission is a perfect AIE-active molecule (Shi et al., 2020b). TBP-1 exhibited high ROS production and antibacterial activities, imaging and monitoring the interplay between AIEgens and bacteria simultaneously. Moreover, with enhanced concentration, TBP-1 significantly improves the antibacterial efficiency against GBS. Thus, the mechanism of antibacterial performance was clarified.

## Materials and Methods

### AIEgen and GBS

AIE photosensitizers (PSs), TBP-1, with an excitation wave of 488 nm, visible light absorption and red fluorescence emission, were granted from Benzhong Tang’s lab, and the chemical structure was shown in Supplementary Figure S1 (Shi et al., 2020). The stock strain of GBS (WC1535) isolated from GIFT tilapia (*Oreochromis niloticus*), China (2015), was applied in our investigation.

### Bacterial Culture

A single colony of GBS was inoculated with a BHI culture medium at 200 rpm, 30°C for 8 h. GBS was determined and adjusted using the turbidimetric method (Biomerieux, France. Coefficient of 1.0 amount to 3.0 × 10^8^ CFU ml^−1^).

### Characterization

Fluorescence spectra of TBP-1 and TBP-1 incubation with GBS (2.1 × 10^8^ CFU ml^−1^) were detected by a Cytation 5 Cell Imaging Multi-Mode Reader (BioTek, United States) with an excitation wave of 488 nm and an emission slit width of 10 nm.

### ROS Detection

The ROS Assay Kit produced by Beyotime (China) was applied to the ROS test according to the instruction. DCFH-DA as a ROS probe could emit fluorescence after oxidized by ROS. The fluorescence of the probe after oxidized by ROS could represent the level of ROS accordingly. The ROS fluorescence intensity of TBP-1 (10 µM), GBS, and GBS (6.0 × 10^8^ CFU) cultured with TBP-1 (1 ml, 10 µM) from 0 to 30 min in the presence and absence of white light irradiation (4.2 mW cm^−2^) was recorded. The ROS fluorescence intensity of GBS (6.0 × 10^8^ CFU ml^−1^) treated with TBP-1 (0, 2, 4, 6, 8, and 10 µM) in the dark or under light irradiation from 0 to 30 min was measured by the Cytation 5 microplate reader (BioTek, United States).

### Antimicrobial Assay

8.1 × 10^8^ CFU of GBS was dispersed into 1 ml PBS containing TBP-1 (0, 2, 4, 6, 8, and 10 µM) and cultured at 200 rpm, 30 °C for 10 min. Subsequently, the GBS was kept in the dark or under white light irradiation (4.2 mW cm^−2^) for 10, 15, and 30 min. After washing and diluting with PBS, 0.1 ml of GBS (about 10^2^ CFU) was spread onto a BHI agar plate. Then, the BHI agar plates with GBS were cultured at 30 °C overnight. Afterward, the number of GBS cultures was counted by images of BHI plates. The number of GBS treated with TBP-1 divided by the CFU of the GBS not treated with TBP-1 gives the survival rate of GBS treated with TBP-1.

### Bacteria Imaging

7.2 × 10^8^ CFU of GBS was treated with 1 ml TBP-1 (10 µM) in the dark or under white light (4.2 mW cm^−2^) for 0 (control), 10, 15, and 30 min. The harvested GBS (at 8000 rpm for 10 min) was incubated with 200 µl propidium iodide (PI) for 20 min. Subsequently, 200 µl of stained GBS solution was washed by PBS twice, and then, 2 µl stained of GBS was used for fluorescence image collection by a confocal laser scanning microscope (ZEISS-LSM800, Germany). Capture conditions: TBP-1: λ_ex_ = 488 nm, λ_em_ = 600–700 nm; PI: λ_ex_ = 543 nm, λ_em_ = 560–620 nm.

### SEM Observation

9.6 × 10^8^ CFU of GBS was suspended in 1 ml PBS or PBS containing TBP-1 (10 µM) at 30°C, 200 rpm, 10 min. Afterward, the GBS suspension was kept in the dark or under white light (4.2 mW cm^−2^) for 10 and 30 min. Subsequently, 30, 50, 70, 80, 90, 95, and 100% ethanol were used for successive dehydration of GBS fixed by glutaraldehyde (2.5%). The GBS samples were coated with gold and then measured by SEM (Regulus 8100 SEM, Hitachi).

### Statistical Analysis

The data of antimicrobial assay were analyzed by IBM SPSS 22.0. The results for all groups were compared using the one-way analysis of variance (one-way ANOVA). The Origin Pro 2018 software was used for graph plotting. In all cases, the significance level of differences was set at *p* < 0.05.

## Results and Discussion

### Characterization of TBP-1

**FIGURE 1:**
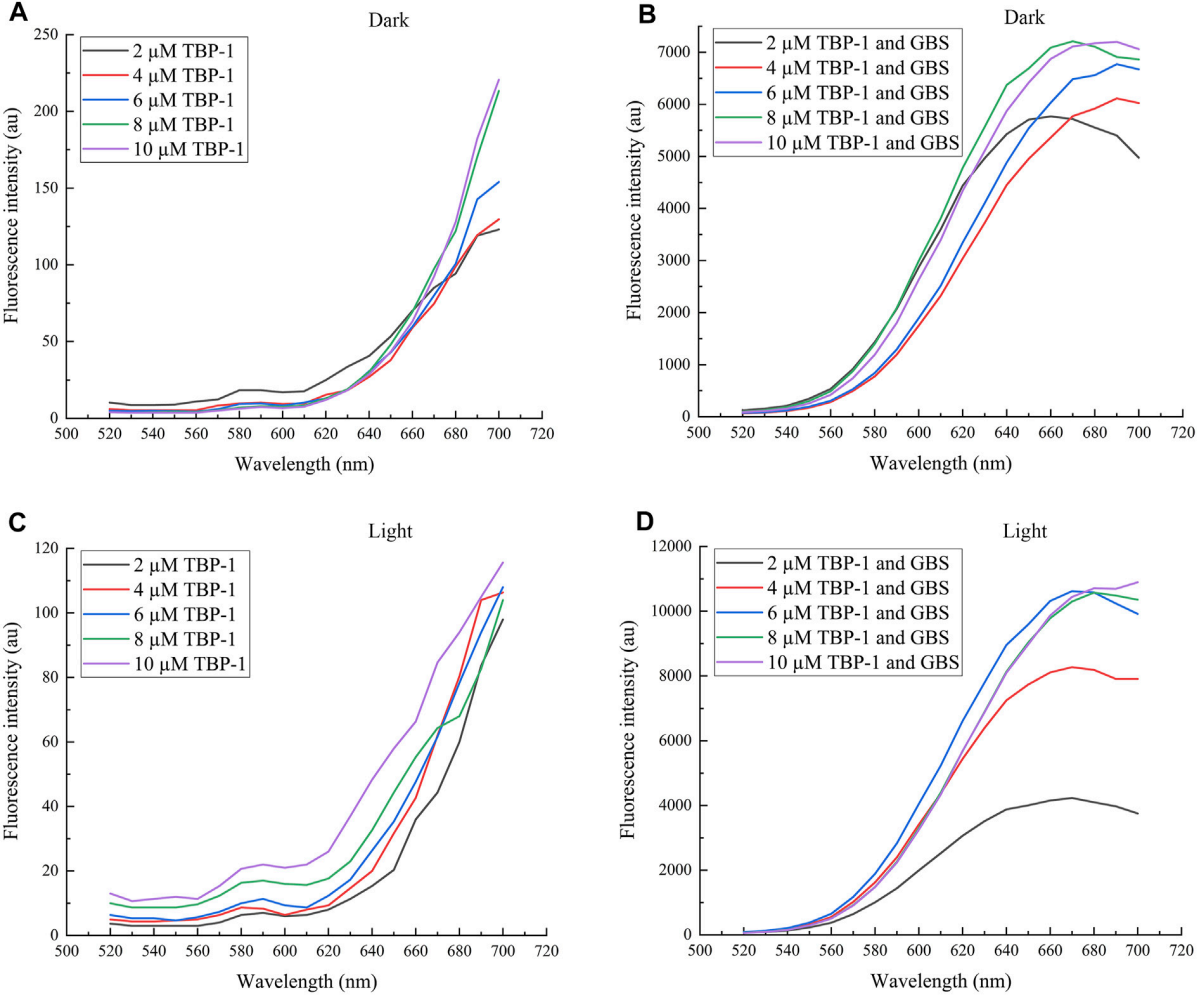
Fluorescence spectra of TBP-1 and GBS incubation with different TBP-1 concentrations in the dark or under white light irradiation for 30 min. **(A)** Different concentrations of TBP-1 in the dark. **(B)** GBS incubation with different TBP-1 concentrations in the dark. **(C)** Different concentrations of TBP-1 with white light irradiation. **(D)** GBS incubation with different TBP-1 concentrations with light irradiation. λ_ex_ = 488 nm.

Fluorescence spectra were used to measure the change in fluorescence intensity of TBP-1 and GBS treated with TBP-1. Compared with TBP-1 (Figures 1A,C), TBP-1 incubation with GBS was observed to have intensive fluorescence emission with peak positions near 670 nm at λ_ex_ = 488 nm (Figures 1B,D). It indicated that TBP-1 exhibits significant AIE effects, especially after light irradiation (Figure 1D). With light irradiation, TBP-1 aggregated on the GBS rapidly actuated by the interaction between GBS and TBP-1. A mass of TBP-1 crossed the GBS membrane into intracellular and caused remarkable changes of fluorescence intensity on account of TBP-1 aggregating within GBS. The concentration of TBP-1 influenced the fluorescence intensity of AIEgen and the incubation with GBS as well. The fluorescence intensity enhanced gradually, accompanied by the increased TBP-1 concentration of AIEgen incubation with GBS and AIEgen alone (Figure 1). Fluorescence emission of TBP-1 changed slightly at different treatment times but decreased after light irradiation (Supplementary Figure S2). However, the fluorescence intensity of GBS increased with increasing TBP-1 concentration and treatment time in the dark or under light (Supplementary Figure S3).

### ROS Generation Ability of TBP-1

**FIGURE 2:**
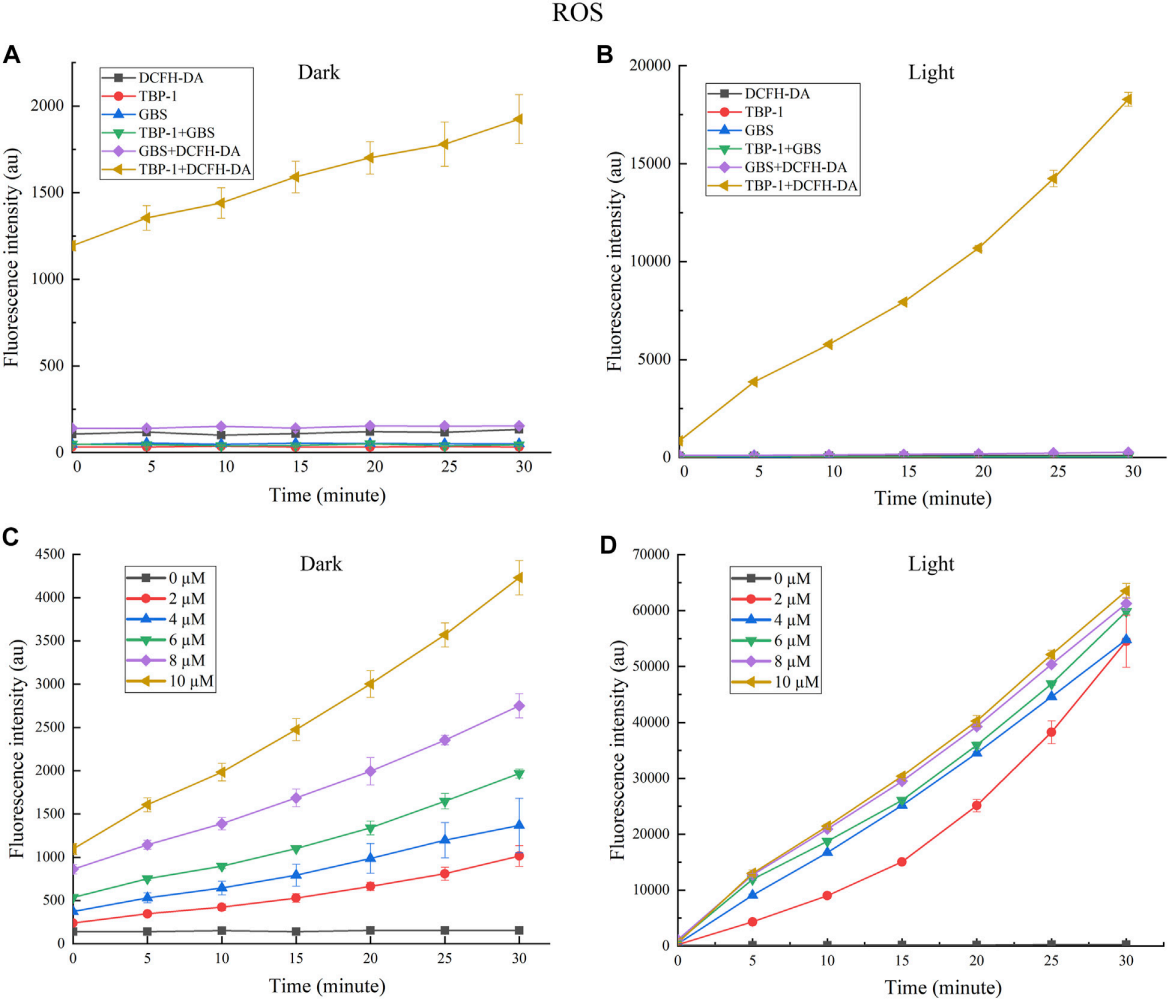
**(A)** Fluorescence intensity at 525 nm generation by TBP-1 (10 µM) and GBS in the dark vs. the irradiation time with or without DCFH-DA (100 µM). **(B)** The fluorescence intensity at 525 nm generation by TBP-1 (10 µM) and GBS under light (4.2 mW cm^−2^) vs. the irradiation time with or without DCFH-DA (100 µM). **(C)** The ROS fluorescence intensity of GBS treated with TBP-1 (0, 2, 4, 6, 8, and 10 µM) in the dark with varying treatment time. **(D)** The ROS fluorescence intensity of GBS added with TBP-1 (0, 2, 4, 6, 8, and 10 µM) exposed to light (4.2 mW cm^−2^) with varying treatment time.

It is generally accepted that lots of antibiotics exhibit ROS-dependent bacteria resistance activity, and PSs based on ROS take a prominent role in the oxidizing milieu (Wainwright, 2012; Xu et al., 2019; You et al., 2017). As a consequence, the ROS generation ability of TBP-1 against GBS in the dark or under light was measured. DCFH-DA was used to research ^1^O_2_ generation with DCFH as a singlet oxygen trapper. It is observed that TBP-1 caused ROS accumulation in a time-dependent manner in the dark or under light (Figure 2). We also observed a higher ROS level of TBP-1 with light irradiation than in the dark, and the ROS generation of TBP-1 enhanced with time (Figures 2A–C). Therefore, the result suggested that TBP-1 as a PS generates ROS in the dark and under light (Figures 2A,B), consistent with previous reports (Li et al., 2014; Uthaman et al., 2020; Zhou et al., 2020). After demonstrating the ROS generation of TBP-1, we detected the ROS levels of GBS incubation with TBP-1 at different concentrations. We observed that TBP-1 caused ROS accumulation with increased dose and time in the dark and under light irradiation (Figures 2C,D). Moreover, an extremely high ROS level of GBS incubation with TBP-1 under the light environment was observed compared to the dark environment (Figures 2C,D). These findings indicate that TBP-1 under light irradiation induces ROS accumulation to kill GBS.

### Antibacterial Studies of TBP-1

**FIGURE 3:**
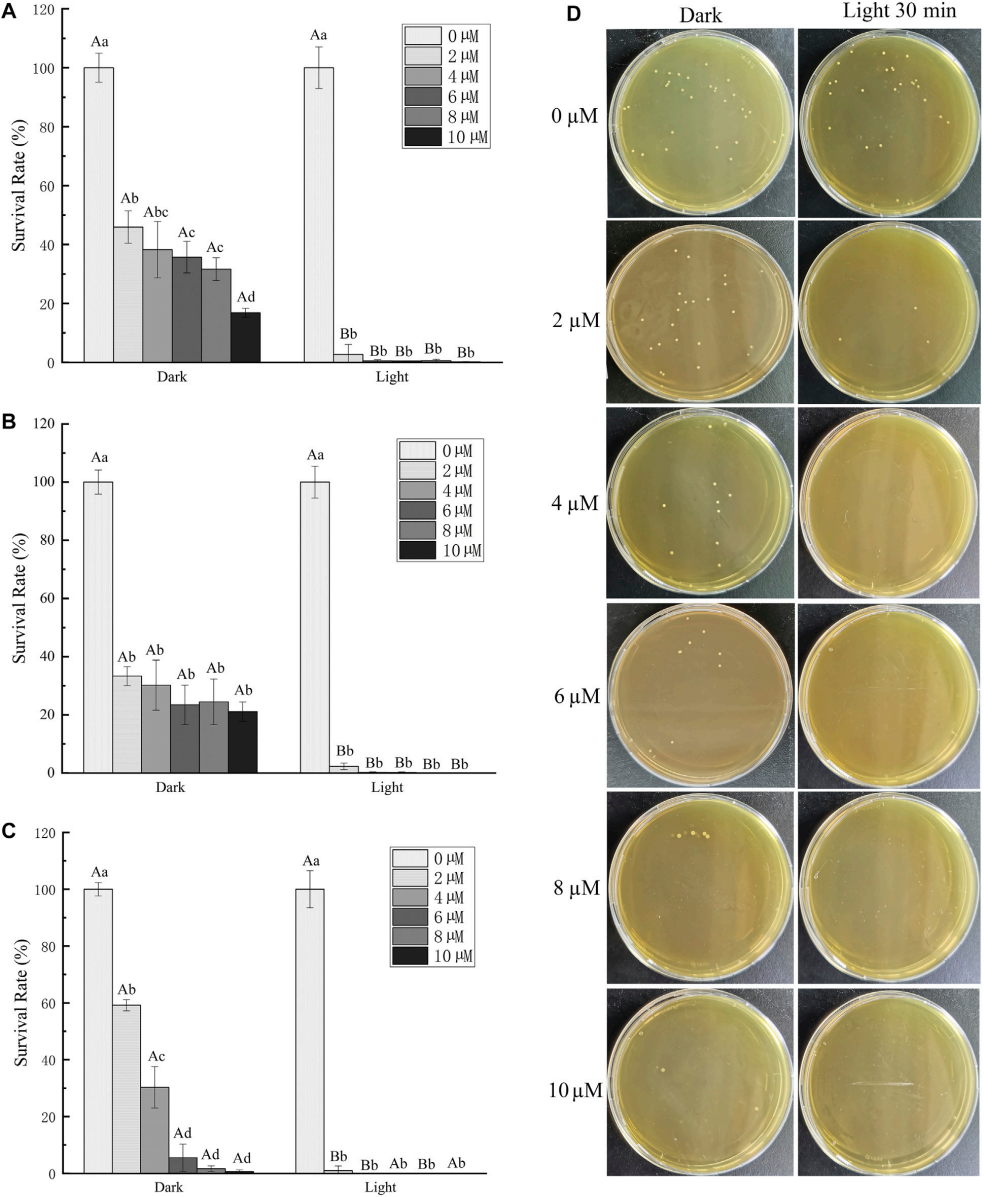
Antibacterial activity of TBP-1 toward GBS. GBS was cultured with TBP-1 at different concentrations for **(A)** 10 min, **(B)** 15 min, and **(C)** 30 min in the dark or under light (4.2 mW cm^−2^). **(D)** Plate photographs of GBS on the BHI agar plate incubated with TBP-1 in the dark or under light irradiation (30 min) at various concentrations. Different lowercase letters indicate significant differences among different concentration groups in the dark or under light (*p* < 0.05). Different uppercase letters indicate significant differences between dark and light exposure at the same concentration (*p* < 0.05).

**FIGURE 4:**
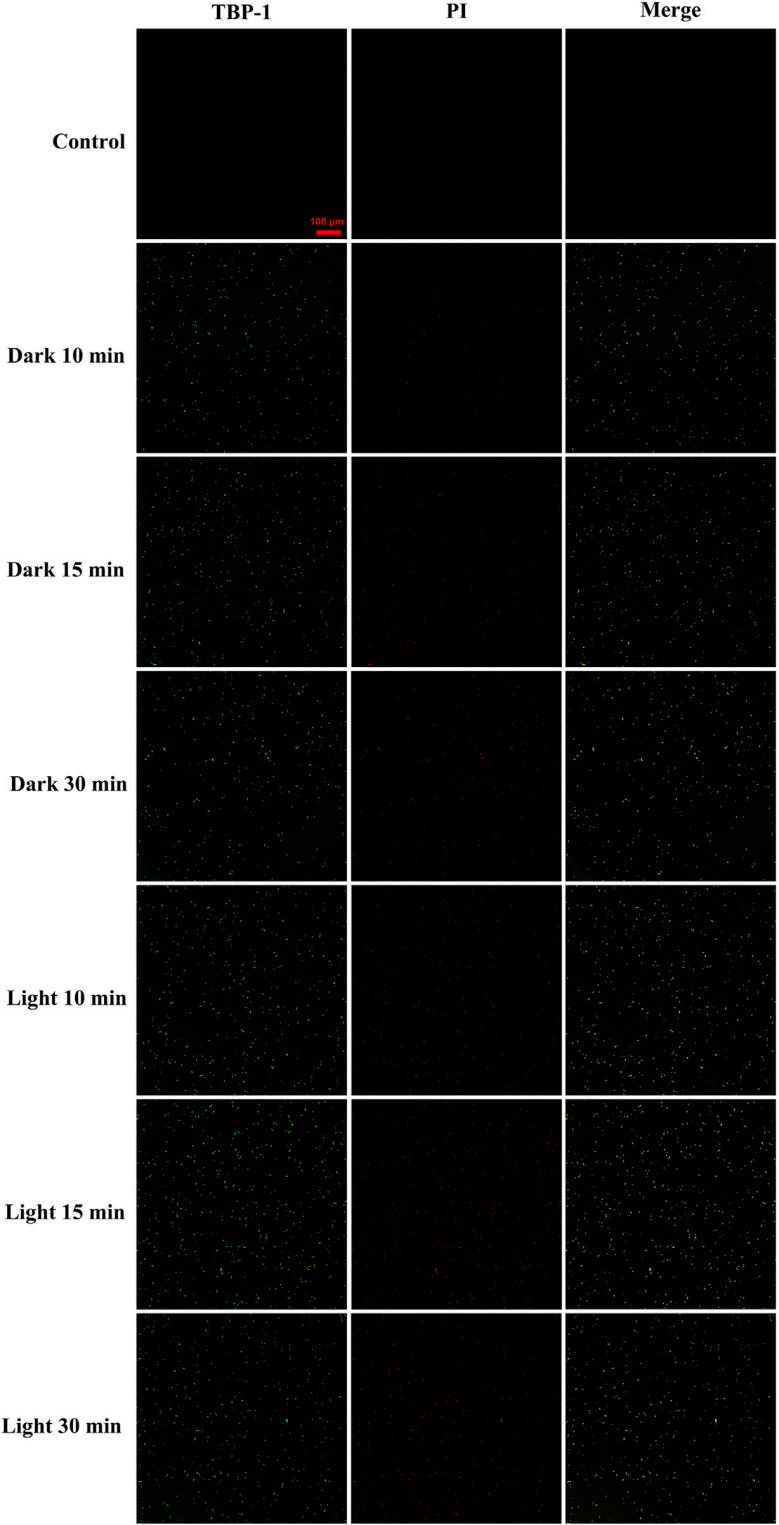
Confocal fluorescent images of GBS after incubation with TBP-1 (10 µM) and PI as a function of time in the dark or under light irradiation. The fluorescence of TBP-1 is exhibited in pseudo green and that of DNA stained by PI in apoptotic GBS is exhibited in red. The merged images were used to check the colocalization. The scale bar is 100 μm. Capture conditions: TBP-1: λ_ex_ = 488 nm, λ_em_ = 600–700 nm; PI: λ_ex_ = 543 nm, λe_m_ = 560–620 nm.

We further studied the antibacterial effect of TBP-1 based on its good ability of ROS production after light irradiation (4.2 mW cm^−2^). GBS, a prominent co-pathogenic bacterium (Crespo-Ortiz et al., 2020), was chosen as the subject bacteria attributed to its serious harm to human, animal, and aquatic products. The antibacterial efficiency of different TBP-1 concentrations (0, 2, 4, 6, 8, and 10 µM) on GBS under the light as a function of time was measured using plate counting (Figure 3). The TBP-1 antibacterial performance on GBS is displayed in Figures 3A–C. TBP-1 showed dark toxicity to GBS, and the antibacterial efficiency was greatly enhanced with light exposure. As exhibited in Figure 3A, the colony counting showed that the survival rates of GBS after incubation at different TBP-1 doses (2, 4, 6, 8, and 10 µM) in the dark decreased to 54.1, 61.7, 64.3, 68.4, and 83.2%, respectively (*p* < 0.05). Moreover, the GBS inhibition surpassed 97.3% after light treatment for 10 min. It is suggested that TBP-1 at a concentration of 4, 6, 8, and 10 µM is potential enough to kill GBS with light irradiation for 10 min, with inhibition effect exceeding 99.5% (Figure 3A). The survival rates of GBS after incubating at 15 min by different TBP-1 doses (2, 4, 6, 8, and 10 µM) in the dark showed a drop of 66.7, 69.8, 76.6, 75.5, and 78.9%, respectively (*p* < 0.05). Moreover, the survival rate decreased by 97.7% with 15 min light irradiation (Figure 3B). Meanwhile, the survival rates of GBS cultured with TBP-1 at different TBP-1 doses (2, 4, 6, 8, and 10 µM) in the dark or under light irradiation for 30 min were counted (Figure 3C). The inhibition effect of different TBP-1 doses (2, 4, 6, 8, and 10 µM) on GBS in the dark was 40.8, 69.7, 94.5, 98.3, and 99.4%, respectively (*p* < 0.05), and the survival rate of GBS after incubation with TBP-1 after light irradiation was less than 99% (Figure 3C). In particular, the bacterial inhibition of GBS with a low TBP-1 dose (4 µM) is more than 99% under light for 10 min (Figure 3A). Moreover, there was scarcely any bacterial colony on the BHI agar plates of GBS with 8 or 10 µM TBP-1 after light irradiation for 15 min (Figure 3B), and 4, 6, 8, or 10 µM TBP-1 followed by 30 min light irradiation (Figure 3C). Plate photographs of GBS on the BHI agar plate incubated with TBP-1 in the dark or under light irradiation (30 min) at various concentrations are shown in Figure 3D. GBS was killed at a lower concentration of TBP-1 (4 µM), and no GBS colony was found on the LB agar plates after treatment with 4, 6, 8, or 10 µM TBP-1 after light irradiation for 30 min. These current research results were consistent with those reported by Hu et al. (2021). The growth and survival rate of bacteria with DPNAP were effectively restrained when exposed to light due to the toxicity induced by ROS (Hu et al., 2021). Therefore, our results certified that TBP-1 exhibited significantly higher phototoxicity on GBS after light irradiation than in the dark.

### Mechanism Under Inhibition of TBP-1 on GBS

The GBS stained by TBP-1 was detected using confocal laser scanning microscope (CLSM) (Figure 4). As a DNA-binding fluorescent dye aimed at the apoptotic cell, PI exhibited red fluorescence. In contrast, TBP-1–stained GBS showed green fluorescence irrespective of intact or damaged membrane. Hence, the extent of GBS membrane damage after TBP-1 incubation could be assessed using red and green fluorescence. Meanwhile, the cell wall and cytoplasm of GBS could be stained with TBP-1 simultaneously, which can be seen from the merged images showing intense interactions between TBP-1 and GBS (Figure 4). Finally, as illustrated in Figure 4, GBS was weakly stained by PI cultured with TBP-1 for 10 and 15 min in the dark, showing almost only green fluorescence. On the other hand, GBS cultured with TBP-1 for 30 min in the dark or 10, 15, 30 min under light irradiation exhibited different extent of red fluorescence. Especially, the GBS cultured with TBP-1 for 10, 15, and 30 min with light irradiation shows brightly green fluorescence, demonstrating intense bacteria damage. Moreover, taking the ROS generation ability (Figure 2) and agar plate count (Figure 3) into consideration, it is suggested that GBS was damaged severely after treatment with TBP-1, especially under light irradiation.

**FIGURE 5:**
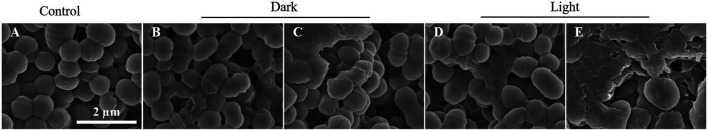
SEM images of GBS incubated with or without TBP-1 (10 µM). **(A)** Control samples of GBS incubation without TBP-1. **(B, C)** GBS incubated with TBP-1 for 10 or 30 min in the dark, respectively. **(D, E)** GBS incubated with TBP-1 under white light (4.2 mW cm^−2^) for 10 or 30 min, respectively. Scale bar: 2 µm.

To further visualize the membrane damage of GBS after TBP-1 treatment, the SEM observation was carried out. In the case of GBS treated with PBS (control groups), GBS with an intact structure was observed (Figure 5A). When cultured with TBP-1 for 10 or 30 min in the dark, collapsed and fused membranes of GBS were imaged (Figures 5B,C). Moreover, we observed that TBP-1 treatment with light irradiation induced severe morphological deformation and accumulation of cell debris (as illustrated in Figures 5D,E) compared to that of the control (Figure 5A) and the dark group (Figures 5B,C). Therefore, TBP-1 showed both dark and light toxicity to GBS, but TBP-1 showed higher antimicrobial activity to GBS with light treatment. SEM is consistent with the results of antibacterial efficiency assessed by the BHI agar plate count (Figure 3) and CLSM (Figure 4).

## Conclusion

In this work, TBP-1 has been proposed to be efficient against GBS through the ROS generated from the photochemical process. TBP-1 can stain and kill GBS in the dark or under light, and its antibacterial activity can further be enhanced under white light irradiation. TBP-1 with low concentration (4 µM) can completely kill GBS under light irradiation. It was detected that TBP-1 produced ROS in the dark or upon exposure to light. TBP-1 displayed excellent biocompatibility toward GBS and showed efficient antibacterial performance through the produced ROS under light irradiation. The mechanism of TBP-1 killing GBS in the dark or under light irradiation was investigated by CLSM and SEM, which imaged the collapsed and damaged membrane of GBS. Our work revealed the excellent antibacterial performance of TBP-1 against GBS. This work will stimulate extensive application of AIE PSs as a novel therapy against pathogenic bacterial infection.

## Data Availability

The original contributions presented in the study are included in the article/Supplementary Material; further inquiries can be directed to the corresponding authors.
