# Supplementary material for: Efficient Inhibition of Streptococcus agalactiae by AIEgen-Based Fluorescent Nanomaterials
Source: Front Chem. 2021 Jul 20;9:715565. doi: 10.3389/fchem.2021.715565 (PMC8329347; doi:10.3389/fchem.2021.715565)
Supplement: Supplementary file 1 [file DataSheet2.docx]

Supplementary Material

## 1 Supplementary Figures


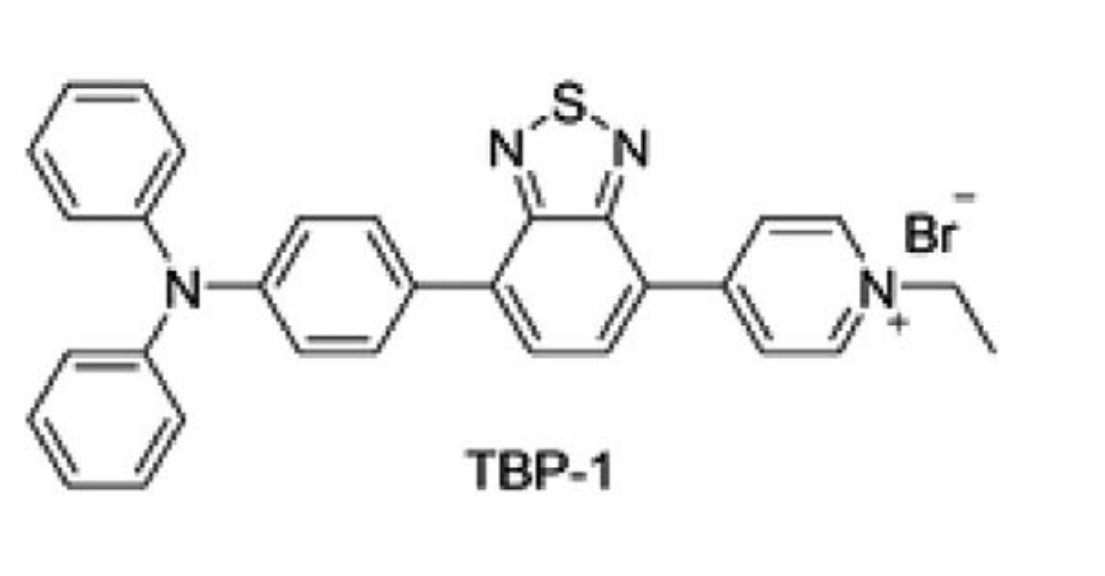


**Supplementary Figure 1.** **The chemical structure of TBP-1 (Shi et al., 2020).**


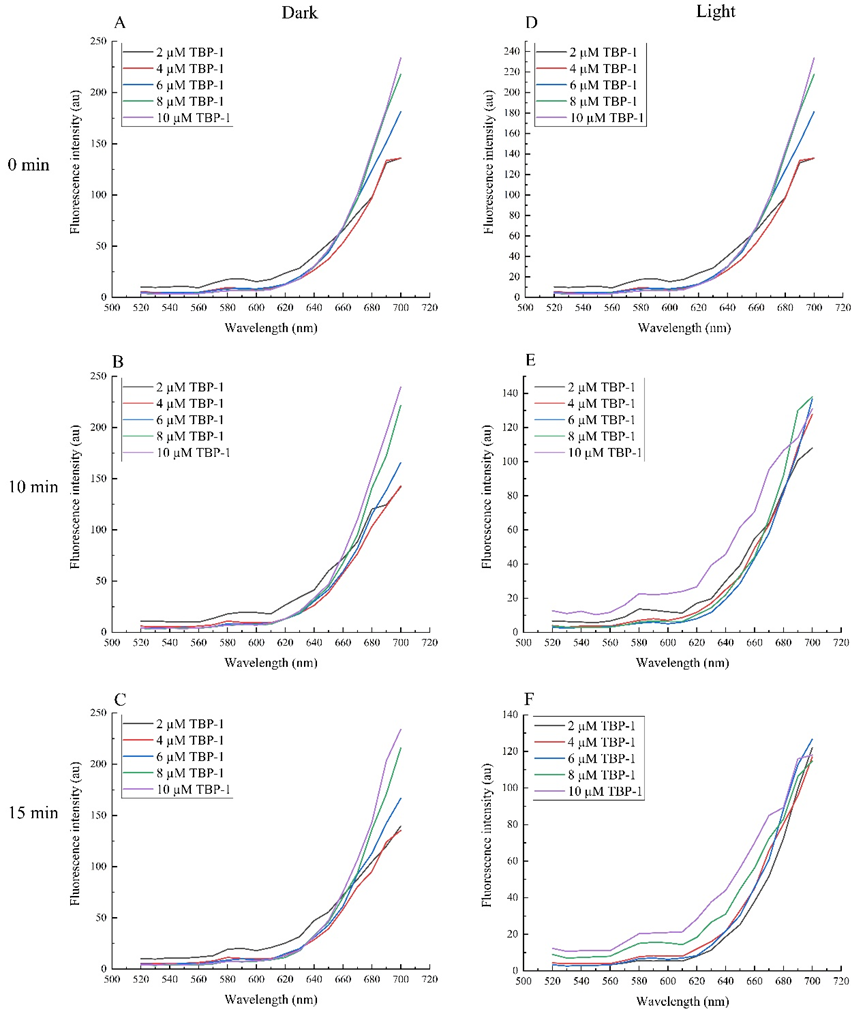


**Supplementary Figure 2.** Fluorescence spectra of TBP-1 with a different concentration in darkness or white light irradiation. **(A, B, C)** Different concentrations of TBP-1 in the dark for 1, 10, and 15 min, respectively. **(D, E, F)** Different TBP-1 concentrations with light irradiation for 1, 10, and 15 min, respectively. λ_ex_ = 488 nm.


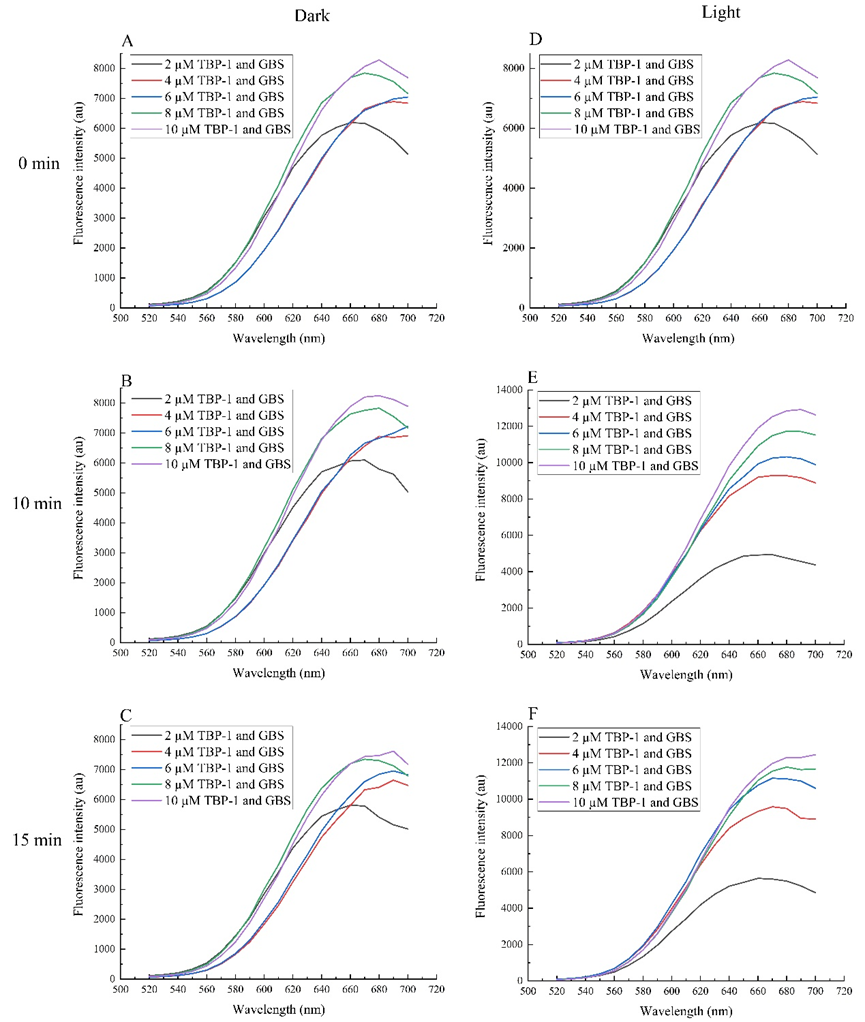


**Supplementary Figure 3.** Fluorescence spectra of GBS incubation with different TBP-1 concentrations in darkness or white light irradiation. **(A, B, C)** GBS incubation with different TBP-1 concentrations under darkness for 1, 10, and 15 min, respectively. **(D, E, F)** GBS incubation with different TBP-1 concentrations with light irradiation for 1, 10, and 15 min, respectively. λ_ex_ = 488 nm.
